# The influence of day/night cycles on biomass yield and composition of *Neochloris oleoabundans*

**DOI:** 10.1186/s13068-017-0762-8

**Published:** 2017-04-22

**Authors:** Lenneke de Winter, Iago Teles Dominguez Cabanelas, Dirk E. Martens, René H. Wijffels, Maria J. Barbosa

**Affiliations:** 1 Bioprocess Engineering, Wageningen University, P.O. Box 8129, 6700 EV Wageningen, The Netherlands; 2 Wetsus-Center of Excellence for Sustainable Water Technology, P.O. Box 1113, 8900 CC Leeuwarden, The Netherlands

**Keywords:** Day/night cycle, Circadian clock, Microalgae, Cell cycle, Biomass composition

## Abstract

**Background:**

Day/night cycles regulate the circadian clock of organisms to program daily activities. Many species of microalgae have a synchronized cell division when grown under a day/night cycle, and synchronization might influence biomass yield and composition. Therefore, the aim of this study was to study the influence of day/night cycle on biomass yield and composition of the green microalgae *Neochloris oleoabundans*. Hence, we compared continuous turbidostat cultures grown under continuous light with cultures grown under simulated day/night cycles.

**Results:**

Under day/night cycles, cultures were synchronized as cell division was scheduled in the night, whereas under continuous light cell division occurred randomly synchronized cultures were able to use the light 10–15% more efficiently than non-synchronized cultures. Our results indicate that the efficiency of light use varies over the cell cycle and that synchronized cell division provides a fitness benefit to microalgae. Biomass composition under day/night cycles was similar to continuous light, with the exception of starch content. The starch content was higher in cultures under continuous light, most likely because the cells never had to respire starch to cover for maintenance during dark periods. Day/night cycles were provided in a ‘block’ (continuous light intensity during the light period) and in a ‘sine’ (using a sine function to simulate light intensities from sunrise to sunset). There were no differences in biomass yield or composition between these two ways of providing light (in a ‘block’ or in a ‘sine’).

**Conclusions:**

The biomass yield and composition of *N. oleoabundans* were influenced by day/night cycles. These results are important to better understand the relations between research done under continuous light conditions and with day/night cycle conditions. Our findings also imply that more research should be done under day/night cycles.

## Background

The circadian clock provides organisms with an internal estimate of the external time. In this way, organisms can program activities at an appropriate time during the day. UV sensitive processes, such as DNA replication, can be scheduled to occur during the night. Such an ‘escape from light’ can provide a fitness benefit to organisms and therefore is thought to be one of the major reasons for the evolution of the circadian clock [1, 2]. Indeed, in plants, it was shown that a substantial photosynthetic advantage was conferred by matching the circadian clock period with that of the external day/night cycle [3]. Cyanobacteria have also shown a competitive advantage of a functioning circadian clock compared to strains with a disrupted clock grown in rhythmic environments [4].

In microalgae, the circadian clock can ‘gate’ cell division to take place during the night [5], while cell growth takes place during the light period. It has been shown that during cell division of synchronous cultures, the biomass yield on light energy was lower than during the rest of the 24-h period [6]. This suggests that light provided during cell division is wasted, leading to a lower photosynthetic efficiency when cell division occurs in the light period. Indeed, it was shown that microalgae make use of their internal starch reserves for cell division, even when division occurs in the light [7]. Therefore, synchronization under day/night cycles would allow cells to grow in size during the day, when light is available, and undergo DNA replication and cell division in the dark, making optimal use of the available light energy [8]. This implies that the circadian clock, that schedules cell division in the night, may also provide a fitness benefit to microalgae by increasing the photosynthetic efficiency.

To verify that, biomass yield is indeed influenced by the circadian clock, also fluctuations in biomass composition need to be considered. Biomass composition is influenced by synchronized cell division and clearly oscillates during a 24-h period [6, 9, 10]. Consequently, biomass yield can be influenced, as for example, more energy is needed for assimilation of 1 g of total fatty acids (TFA) than for assimilation of 1 g of starch.

A possible influence of the circadian clock on biomass yield and composition would have implications for research on microalgae, since it might not be possible to translate research from continuous conditions in the lab to outdoor conditions. A lot of research on microalgae is done under continuous light conditions [11–14]. Research focussed on simulating outdoor conditions with light/dark cycles is done by providing the light in ‘block’ form, i.e. light is on/off [15] or ‘sine’ wave form, i.e. slowly on/off [16]. However, a solid comparison between cultures grown under continuous light and cultures grown under day/night cycles is lacking, as well as a comparison between cultures grown under ‘sine’ and block’ lighting regimes.

The aim of this research was to investigate the influence of day/night cycles on microalgal biomass yield and composition. Therefore, *Neochloris oleoabundans* was grown in a continuous turbidostat photobioreactor under 3 different regimes: continuous light, 16:8 day/night cycles (16D8N) as a “block” and 16:8 day/night cycles (16D8N) as a “sine”. Biomass growth, oxygen production and biomass composition were monitored and compared among all 3 experiments to compare synchronized and non-synchronized cultures. In this way, the influence of day/night (D/N) cycles on biomass yield and composition was revealed.

## Results and discussion

### Growth under different light regimes

Steady state culture of *N. oleoabundans* was reached by keeping the light absorbed constant (turbidostat). During steady state, the daily dilution rate is equal to average specific growth rate over a day (Eq. 1). However, as in turbidostat cultures, the light absorbed is kept constant, slight changes in biomass concentration are possible during the light period due to changes in biomass composition (Eq. 2). As a result, dilution rate does not translate into growth rate in the cultures under 16:8 day/night cycles (16D8N cycles), where oscillations in biomass composition occur that are not observed under continuous light. Therefore, the term dilution rate will be used instead of specific growth rate.

**Fig. 1:**
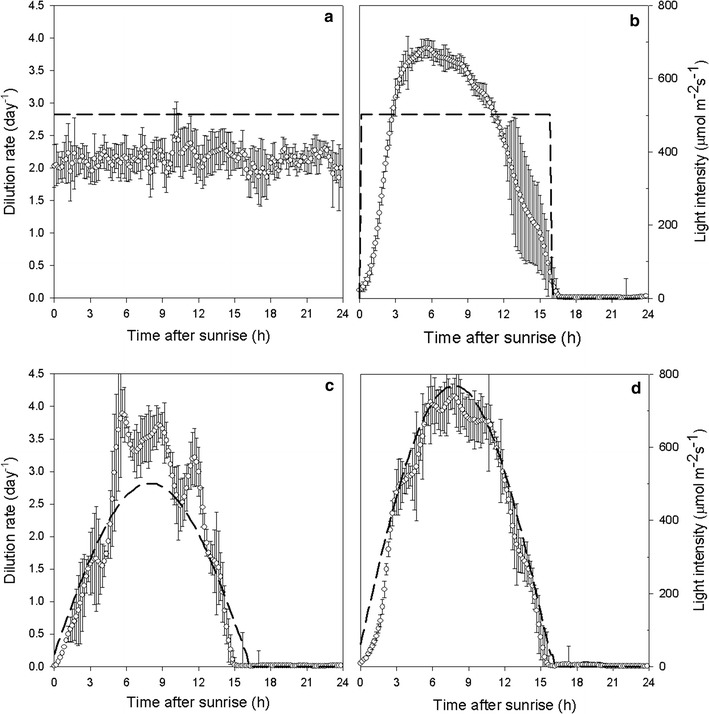
Daily variation in dilution rate in steady state in cultures grown under continuous light (**a**), 16D8N cycles applied in block form (**b**), sine with low light intensity (LL) (**c**) and sine with high light intensity (HL) (**d**). Dilution rate was averaged over at least 5 days in steady state and *error bars* represent standard deviation between those days. Ingoing light intensity is plotted in *dashed lines*

**Fig. 2:**
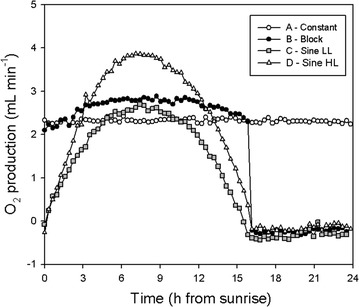
Oxygen production rate (Φ_O2,prod_) during a day in steady state for experiments A (*white circles*), B (*black circles*), C (*grey squares*) and D (*white triangles*)

The dilution rate was calculated from the amount of overflow produced using 1-h intervals and is plotted in Fig. 1. Table 1 presents the light conditions for each experiment, which can be used to understand the relation between Table 1 and Fig. 1. The light intensity applied to the culture is shown in Fig. 1 to assist data interpretation (dashed line, plotted on the secondary vertical axis). The average 24-h dilution rate was 2.2 [0.02], 1.7 [0.01], 1.4 [0.02] and 1.7 [0.02] for experiments A, B, C and D, respectively (standard errors are indicated in brackets). In the experiments done under a 16D8N cycle (see Figs. 1b, 2c, d), the dilution rate varied from 0 day^−1^ in the night till approximately 4 day^−1^ during the day. The maximum dilution rate was slightly higher in experiment D, where maximum light intensity applied to the culture was the highest. Since the light intensities used in the experiments were below the photoinhibition point (data not shown, from previous experiments), the maximum dilution rate was dependent on incident light intensity.

**Table 1:** Light conditions used in the experiments

Experiment	Photoperiod (h)	Light supply	Max intensity (μmol m^−2^ s^−1^)	PF (mol L^−1^ day^−1^)
A	24	Continuous	500	1.96
B	16	Block	500	1.30
C	16	Sine	500	0.86
D	16	Sine	770	1.30

All cultures grown under D/N cycles showed similar daily fluctuations in growth, regardless the light regime. In all D/N cycle experiments, the dilution rate increased until approximately 6 h after ‘sunrise’ and started to decrease after 9 h after ‘sunrise’. Strikingly, also in experiment *B*, where light was applied at constant intensity (as a ‘block’) during the day period, dilution rate more or less followed a sine curve. The maximum in dilution rate between 6 and 9 h of light suggests that a timing mechanism is involved in growth of *N. oleoabundans*. The total amount of light received by the cultures in experiment B, C and D was different before reaching the maximum dilution rate. Therefore, the attainment of the maximum dilution rate was not a function of the amount of light received, and more likely resulted from a process timed by the circadian clock. Photosynthesis is well known to be under control of the circadian clock [17], and daily variations in photosynthetic rate were already described in earlier research [18, 19].

To see if the maximum dilution rate coincided with a maximum in photosynthesis rate, the gas flow coming from the reactor was analysed using a mass spectrometer. In Fig. 2, the oxygen production rates in mL min^−1^ during a 24-h cycle in steady state are shown. As it can be seen, oxygen production rates in experiments C and D closely follow a sine curve, indicating that the culture as a whole was growing light limited with higher light intensities leading to higher oxygen production rates [20]. As such a maximum oxygen production rate was expected when ingoing light intensity (PFDin) was at its top. However, in experiment B, where light intensity was kept constant for a 16-h period, a maximum oxygen production rate was also obtained (although the difference between the beginning of the day and the maximum was much smaller than in experiment C, for example). In combination with the strong sine that was observed in dilution rate (Fig. 1b), this again suggests the influence of the circadian clock, which synchronizes cell division during the night in the 16D8N cycle [21].

The synchronized cell division influences biomass composition [6], which can in turn influence dilution rate in the turbidostat (Eq. 2). An interesting observation is that the increase in oxygen production rate in experiment B was slower than the increase in dilution rate (Figs. 1b, 2). This means that growth rate increased immediately when light was turned on and therefore the dilution rate lagged behind. Also the decrease in dilution rate after the maximum was steeper than the decrease in oxygen production rate. This indicates that after the maximum in dilution rate the absorption cross section (a) of the biomass decreases, again meaning that other biomass constituents, like starch, were synthesized at higher rates than the light absorbing material.

Under the continuous white LED light conditions in experiment A, all timing processes were clearly lost or randomized (Fig. 1a), resulting in an average dilution rate of 2.2 day^−1^. This was expected when applying a constant intensity of white LED light, as a synchronized cell cycle can not be maintained under these conditions [6]. Also, oxygen production rate in experiment A was constant, at 2.3 mL min^−1^. In experiment B, where light was supplied with the same intensity as in experiment A, oxygen production rate during the day was higher with a maximum oxygen production rate of 2.8 mL min^−1^. When biomass composition in experiment A and B is similar, the higher oxygen production rate already indicates a higher net photosynthetic efficiency during the day in experiment B, as more oxygen was produced using the same amount of photons. Such observation can be confirmed by the twofold increase in the photosynthetic efficiency of experiment B (Table 2). In “Biomass yield and productivity”, the biomass yield and productivity of the synchronized cultures (experiments B, C, D) and the randomly dividing culture (experiment A) will be further addressed.

**Table 2:** Respiration rates and photosynthetic efficiency for all 4 experiments

	Respiration rates			Photosynthetic efficiency
	mmolO_2_/h	g/L/h	g/g/h	%
A-constant	–	–	–	0.96
B-block	−0.63	−0.01	−0.01	1.98
C-sine LL	−0.87	−0.01	−0.02	2.17
D-sine HL	−0.43	0.00	−0.01	0.99

### Biomass composition

**Fig. 3:**
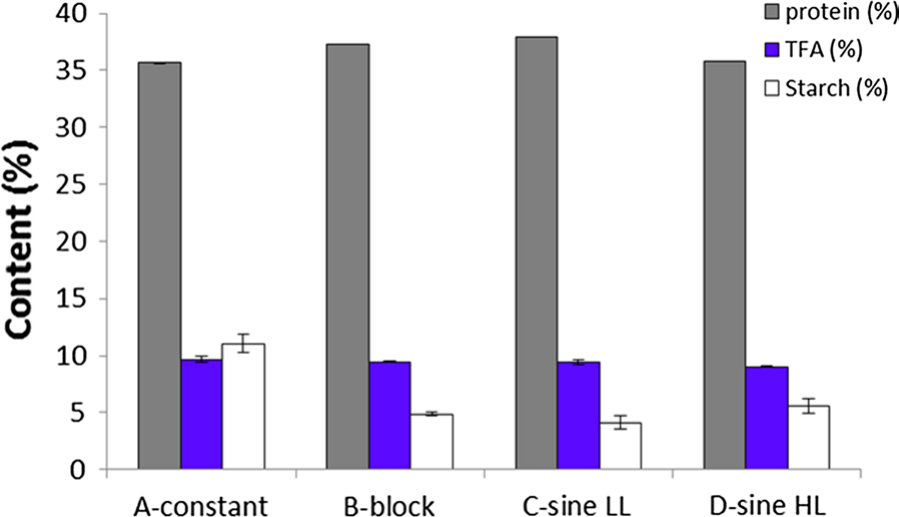
Biomass composition of *N. oleoabundans* with different lighting regimes as a percentage of dry weight. Biomass was pooled over the 24-h cycle and *error bars* represent the variability (max–min/2) between triplicate 24-h cycles

Biomass yield on light can also be influenced by biomass composition, as will be further discussed in “Biomass yield and productivity”. Therefore, major biomass constituents of the harvested biomass were determined. Samples were taken from the overflow of 3 subsequent days in steady state and analysed for protein content, TFA content and starch content. In Fig. 3, the results are presented in % of DW and error bars represent the variability [(max–min)/2] between the 3 days of steady state. Protein content and TFA content were the same in all experiments. However, starch content was twice as high in the continuous light culture. This result can be explained because starch is built up during the day and respired during the night to cover for dark respiration of biomass [22]. During a 24-h period under day/night cycles, biomass composition clearly oscillates [10]. This has to be kept in mind when translating research on the biomass composition of microalgae done under continuous light conditions in indoor experiments to outdoors [11, 12]. For *N. oleoabundans* grown in nutrient replete conditions, the only difference was observed in starch content. However, it should be noted that the sum of the measured biomass constituents only accounts for approximately 50% of total dry weight, which means that other biomass constituents can also show differences. Previous research with *N. oleoabundans* indicates a value of total carbohydrates of around 50%. If we consider that starch is part of total carbohydrates, we can consider approximately 40% of putative carbohydrates [11, 23]. With that in mind, we can consider our biomass analyses to cover for up to 90% of components. The 10% left can include the ash content and nucleic acids, closing the gap to the full biomass composition. In addition, under other process conditions or in other microalgae also differences in TFA might be expected between continuous light and day/night cycle cultures, as also fatty acids might be used in respiration [24].

There was no difference in protein, starch and TFA content between experiment B and experiment C and D, which indicates that providing light energy in block is a good alternative for using sine forms when working with D/N cycles in the laboratory.

### Biomass yield and productivity

**Fig. 4:**
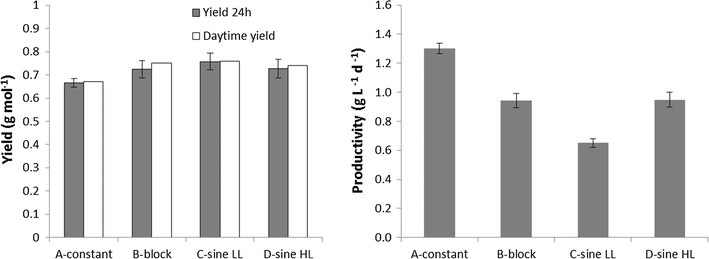
Yield and productivity of the cultures grown under continuous light and 16D8N cycles applied in block, sine LL and sine HL. *Grey bars* represent productivity and yield calculated over 24 h, using the biomass harvest of 3 days and *error bars* represent the variability (max–min/2) between triplicate measurements. *White bars* represent yield calculated over just the daytime period, using the O_2_ production rates

Biomass yield in grams of DW per mole of photons was calculated from Eq. 4 and plotted in grey bars in Fig. 4 (left), with error bars representing the variability [(max–min)/2] between three subsequent days in steady state. The biomass yield in continuous light was lower (approximately 10 ± 1%) than the biomass yield in the D/N cycle experiments. Furthermore, in the D/N cycle experiments, a negative oxygen production rate was measured during the night, indicating that oxygen was consumed through respiration of biomass (Fig. 2, Table 2). Therefore, in the D/N cycle cultures (experiments B, C, D, Table 2), some biomass is lost during the night, which is a known biological response since the cells need to respire part of the biomass to cover their maintenance requirements [25]. As such, the biomass yield on light during the daytime period could be even higher than the value measured here over a whole day.

To confirm this, the biomass yield on light during the light period (16 h) was calculated (Eqs. 6, 7, 8, 9), by integration of the oxygen production rates in Fig. 2 and converting this to biomass production with the stoichiometric growth equation for growth of *N. oleoabundans* on nitrate. The results are plotted as white bars in Fig. 4 and referred to as daytime yield. As expected, the daytime yield in the D/N cycle cultures calculated from the oxygen production rates was higher than the 24-h yield, whereas no difference was found in the continuous light experiment. The difference in biomass yield between continuous light and D/N cycle cultures was approximately 15%. The increased biomass yield in the D/N cycle cultures is not due to the lower starch content in these cultures. Light energy was used 10–15% more efficiently during the day in the synchronized cultures, where cell division occured during the dark period, than in the continuous light culture, where cell division occured randomly. A possible explanation for the lower yield in the continuous light culture is that during cell division light is used less efficiently for a certain period of time. Possibly, cells use starch as an energy source for cell division in the light, like they also do during the night [7]. Therefore, some of the light energy provided during cell division might be wasted.

However, as the difference in biomass yield was only 10–15%, biomass productivity was most likely mainly dependent on the amount of light provided to the culture. In Fig. 4 (right figure), it can be seen that productivity was exactly the same in the experiments with light provided in a block (B) and in a sine (HL) (D). Those two experiments received the same amount of photons, whereas the continuous light culture (A) received more, and the sine LL (C) culture received less. So, productivity was mainly a function of the amount of photons received and therefore providing more light will result in a higher productivity.

Interestingly, no difference in yield was observed between the experiment where light was provided in a block of constant light intensity (B) and the experiments where light was provided in a sine form (C and D). It was expected that when light was provided in lower intensities in the beginning of the day, algae had more time to adapt to the increasing light intensity and therefore would be able to use the light more efficiently [20]. Indeed, in experiment C, where light intensities were the lowest, yield was higher. In experiment D, where light at the maximum was the highest, yield was lower. However, the obtained differences were within the measurement error, which means that under our experimental conditions providing light in a block in indoor experiments can provide a good and easy to operate alternative to providing light in sine form.

## Conclusions

Microalgae cultures of *N. oleoabundans* that were synchronized by day/night cycles were able to use the light provided 10–15% more efficiently than cultures grown under continuous light. In other words, the net efficiency of light usage varies over the cell cycle and the ability to schedule cell division during the night provides a fitness benefit to microalgae. Protein, TFA and starch contents of the 16D8N cycle cultures were the same. However, a higher starch content was found when continuous light was provided. The microalgae under these conditions never had to spend starch for respiration during a dark period, and therefore starch content remained high. No difference in biomass yield and composition was found when the light during the 16D8N cycle was provided in a block or in a sine. Therefore, providing light intensity in a block could be a reliable and easy to operate alternative for using sinuses when working with D/N cycles with *N. oleoabundans* under the boundaries of our current experimental conditions.

In conclusion, the biomass yield and composition of *N. oleoabundans* were influenced by the circadian clock when grown under D/N cycles. These results could be used to translate research done under continuous light conditions to outdoor D/N cycle conditions. For example, the effect of D/N cycles could be used to time harvesting to periods of higher yields of biomass or products. To fully control an outdoor facility taking advantage of D/N cycles would require further research at the same location to first assess cell synchronization and then applying the knowledge to possibly time biomass harvesting. However, the results here presented drawn attention to the dynamic nature of microalgae culture and the impact that it might have on biomass yields and productivities.

## Methods

### Preculture

*Neochloris oleoabundans UTEX 1185* (The culture collection of Algae, University of Texas, Austin) was cultivated in 250 mL shake flasks containing 100 mL adjusted BBM medium with pH 7.5 [26] on a shaking incubator (Max Q 3000, Barnstead) at 120 RPM at a temperature of 25 °C. Light was provided at an intensity of 20–40 μmol m^−2^ s^−1^ through 16D:8 N cycles. Four days prior to inoculation, the cultures were transferred to a second shaking incubator at 120 RPM (Orbital Incubator, Sanyo, Japan) with light provided continuously at an intensity of 150 μmol m^−2^ s^−1^. Temperature was again kept at 25 °C and the headspace of the incubator was enriched with 5% CO_2_.

### Photobioreactor set-up and experimental conditions

**Fig. 5:**
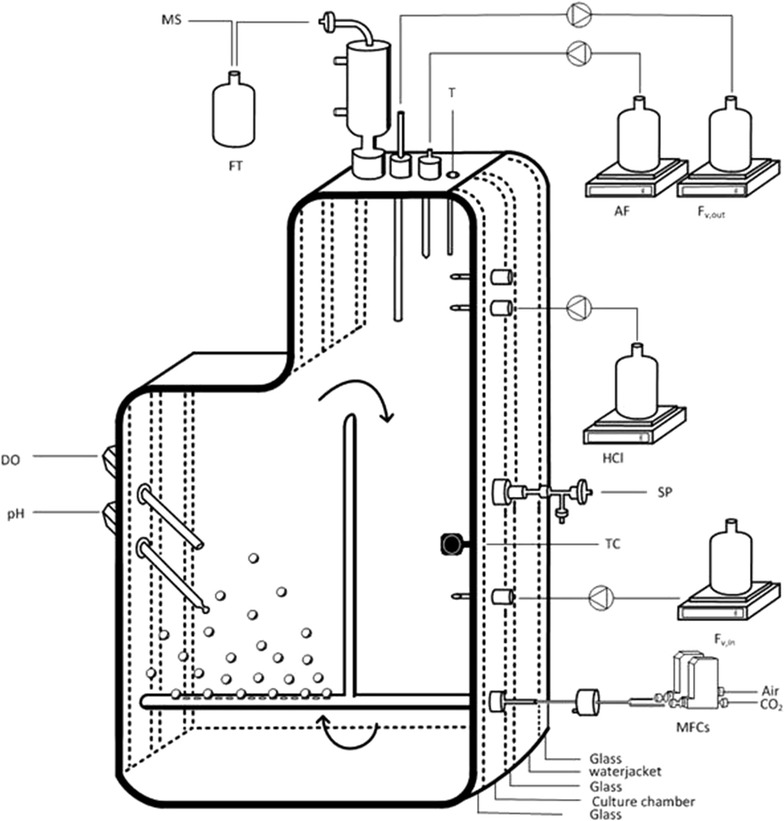
Schematic overview of the PBR set-up. *MS* mass spectrometer, *AF* antifoam, *Fv,out* overflow vessel on balance, *HCl* pH control, *T* temperature sensor, *SP* sample port, *TC* light sensor for turbidostat control, *Fv,in* medium inflow, *MFCs* mass flow controllers, *DO* dissolved oxygen sensor, *pH* pH sensor

*Neochloris oleoabundans* was continuously cultivated in a flat panel photobioreactor (PBR) (Labfors 5 Lux, LED Flat Panel Option, Infors HT, Switzerland) (full description of the system is available at Breuer et al. [11]). A schematic overview of the PBR set-up is shown in Fig. 5. The light path of the PBR was 20 mm and the working volume was approximately 1.8 L. The light was supplied by 360 LEDs (warm white light, spectrum from 450–620 nm). A black cover was placed on the back of the reactor to ensure that no environmental light was able to enter the reactor. In addition, a cover especially designed to fit the PBR was placed in between the LED panel and the reactor, functioning as a light tunnel and again preventing environmental light from entering the PBR. Ingoing and outgoing light intensities (PDF_in_ and PDF_out_) were measured on the surface using a Li-cor quantum sensor (LI250 light metre, LI-COR, USA).

After inoculation, PDF_in_ was gradually increased until it reached the final set point, in order to allow the microalgae to adapt to the new light conditions. When PDF_out_ reached its final setting due to biomass growth, the light regime was changed to the final light settings. Table 1 shows a summary of the different light settings that were tested. The maximum light intensity in experiment D was chosen such, that the total amount of photons provided to the algae was the same as in experiment B. In experiment C, the maximum light intensity of the sine was the same as the maximum light intensity in experiment A and B.

The temperature inside the PBR was maintained at 30 °C using the internal temperature control system, connected to the water jacket of the PBR. Water was provided to the Infors HT system at a constant temperature of 20 °C through the use of an external cryostat (RE306/E300, Lauda, Germany). The pH was maintained at 7.5 ± 0.2 by an automatic supply of 1 M HCl. Dissolved oxygen was measured online and foam formation was prevented by manually supplying, every morning and afternoon, a 2%_(v/v)_ antifoam solution (Antifoam B^®^ silicone emulsion, Mallinckrodt Baker B.V., Deventer, The Netherlands). The culture was continuously sparged with 1000 mL min^−1^ air enriched with 2%_(v/v)_ of CO_2_, provided by the set of mass flow controllers embedded in the Infors HT system. The air was leaving the reactor through a condenser, which was connected to a cryostat at 2 °C, to prevent culture evaporation.

When light was on, also the turbidostat control was turned on. Turbidostat control ensured dilution of the culture with fresh culture medium when PDF_out_ dropped below the set value. PDF_out_ was set as 10% of PDF_in_ (this holds also for sinus functions, which has different set-points during the day). In this way, the light absorbed by the culture was always 90% of PDF_in_ (this choice was made due to the operational mode as turbidostat, which were regulated by the light sensors incorporated in the system). The system was allowed to reach steady state, which was defined as a constant biomass concentration measured at the same time every day, and a constant average daily dilution rate for a period of at least 3 residence times. In the steady state, the daily growth rate (μ_24_) is equal to the dilution rate (D), which was monitored by logging the amount of overflow produced (V_24_) over 24 h (see Eq. 1).

1 $$ \mu_{24} = D = \frac{{{\text{V}}_{ 2 4} }}{V}. $$

The total amount of absorbed light was kept constant by the turbidostat control, which means that changes in biomass concentration in the reactor are possible when light absorbing properties of the biomass change. Therefore, growth rate does not equal the dilution rate over smaller time intervals during the day, but becomes a function of biomass growth rate (*μ*) and the change in absorption cross section (a) of the biomass (Eq. 2). Derivation of Eq. 2 can be found in the "Appendix".

2 $$ D = \mu + \frac{1}{a}\frac{{\text{d}a}}{{\text{d}t}}. $$

### Sampling, biomass analysis and calculation of yield from overflow

Samples were taken daily at the same time to monitor biomass growth and steady state by measuring the optical density at 750 and 680 nm (OD_750_ and OD_680_), cell number, cell size, total cell volume and dry weight (DW), as described by Kliphuis et al. [13]. During steady state, the total overflow of a 24-h cycle was collected and kept on ice. This was done for 3 consecutive cycles. From the harvested biomass, DW samples were taken to determine biomass concentration (*C*_x_) and calculate biomass productivity (*P*_x_) and yield (*Y*_E/x_) over 24 h according to Eqs. 3 and 4.

3 $$ P_{\text{x}} = \mu_{ 2 4} \cdot C_{\text{x}}, $$

4 $$ Y_{x/e} = \frac{{\mu_{24} \cdot C_{\text{x}} }}{PF}, $$

in which PF is the photon flux. The remainder of the biomass was centrifuged at 4500 rpm for 30 min. The pellets were washed with demi water and again centrifuged at 4500 rpm for 20 min. Pellets were then stored at −20 °C until freeze drying. After freeze drying, pellets were grinded with a mortar and pestle and subsequently aliquots of the biomass were used to determine major biomass constituents (proteins, starch and fatty acids) as described by de Winter et al. [6].

### Gas analysis and calculation of yield from O_2_ production

Outgoing air flow was analysed with a Prima dB mass spectrometer (Thermo Fisher Scientific). Oxygen production rate (*Φ*_O2,prod_) in mL min^−1^ was calculated according to Eq. 5:

5 $$ \phi_{02,prod} = \phi_{N2,b} \cdot \left( {\frac{{x_{02} }}{{x_{N2} }}} \right)_{out} - \phi_{02,b}, $$

in which *Φ*_N2,b_ and *Φ*_O2,b_ are the nitrogen and oxygen flow in mL min^−1^ measured in a baseline prior to inoculation of the reactor. *x*_O2_ and *x*_N2_ are the fractions of oxygen and nitrogen in the outgoing gas, and the fraction of nitrogen is assumed constant. From this, the oxygen production rate (OPR) and CO_2_ consumption rate (CUR) in mmol h^−1^ were calculated:

6 $$ OPR = \frac{{\varphi_{O2,prod} \cdot p_{0} }}{{R \cdot T_{0} }} \cdot 0.06, $$

7 $$ CUR = \frac{OPR}{PQ}, $$

in which PQ is the photosynthetic quotient, which is 1.42 according with Pruvost et al. [27]. Following we have the stoichiometric growth equation for growth of *N. oleoabundans* on nitrate (Eq. 8), in which molecular composition of *N. oleoabundans* biomass was taken from Pruvost et al. [27].

8 $$ {\text{CO}}_{ 2 } + \, 0. 9 3 {\text{ H}}_{ 2} {\text{O }} + \, 0. 1 5 {\text{ NO}}_{ 3} \to {\text{ CH}}_{ 1. 7} {\text{O}}_{0. 4 3} {\text{N}}_{0. 1 5} + { 1}. 4 {\text{ O}}_{ 2} + \, 0. 1 5 {\text{ OH}}. $$

Finally, with the molecular weight of *N. oleoabundans* biomass, *m*_biomass_, which is 23.45 g C-mol^−1^ [27], the biomass yield on light (*Y*_X/e_) in g mol^−1^ was calculated (Eq. 8) to be compared with the yield calculated in Eq. 3.

9 $$ Y_{x/e} = \frac{{CUR \cdot m_{biomass} }}{PF}. $$

## Appendix

Derivation of Eq. 2

Balance over the amount of absorbing material a (m^2^.gDW^−1^):

$$ V\frac{{\text{d}(C_{x} \cdot a)}}{{\text{d}t}} = V \cdot (a \cdot \frac{{\text{d}C_{x} }}{{\text{d}t}} + C_{x} \cdot \frac{{\text{d}a}}{{\text{d}t}}) = V \cdot r_{a} \cdot C_{x} - F \cdot C_{x} \cdot a = 0, $$

$$ D = \frac{{r_{a} }}{a}, $$

$$ \frac{{\text{d}C_{x} }}{{\text{d}t}} = - \frac{{C_{x} }}{a} \cdot \frac{{\text{d}a}}{{\text{d}t}}. $$

Dry weight balance:

$$ V\frac{{\text{d}C_{x} }}{{\text{d}t}} = V \cdot \mu \cdot C_{x} - F \cdot C_{\text{x}} = 0, $$

$$ D = \mu - \frac{1}{{C_{x} }}\frac{{\text{d}C_{x} }}{{\text{d}t}}. $$

Combining both balances gives:

$$ D = \mu + \frac{1}{a}\frac{{\text{d}a}}{{\text{d}t}}. $$

## Abbreviations

TFA: total fatty acids
D/N: day/night
PFD_in_: ingoing light intensity μmol m^−2^ s^−1^
PFD_out_: outgoing light intensity μmol m^−2^ s^−1^
*μ*_24_: daily average specific growth rate Day^−1^
*μ*: specific growth rate day^−1^
*D*: dilution rate day^−1^
*a*: absorption cross section m^2^ gDW^−1^
*V*_24_: volume overflow collected over 24 h L h^−1^
*V*: volume of bioreactor L
*C*_x_: biomass concentration g L^−1^
*P*_x_: productivity g L day^−1^
*Y*_x/e_: yield of biomass on light g mol^−1^
PF: photon flux mol L^−1^ day^−1^
*Φ*_O2,prod_: oxygen production rate mL min^−1^
*Φ*_N2,b_: nitrogen flow in baseline mL min^−1^
*Φ*_O2,b_: oxygen flow in baseline mL min^−1^
x_O2_: fraction of oxygen in outgoing gas
x_N2_: fraction of nitrogen in outgoing gas
*P*_o_: ambient pressure Pa
*R*: gas constant (Pa L^−1^)/(mmol K^−1^)
*T*_0_: absolute temperature K
OPR: oxygen production rate mmol h^−1^
CUR: carbon dioxide consumption rate mmol h^−1^
PQ: photosynthetic quotient
*m*_biomass_: molecular weight of the biomass g
